# Bottom-Up Catalytic Approach towards Nitrogen-Enriched Mesoporous Carbons/Sulfur Composites for Superior Li-S Cathodes

**DOI:** 10.1038/srep02823

**Published:** 2013-10-02

**Authors:** Fugen Sun, Jitong Wang, Huichao Chen, Wenming Qiao, Licheng Ling, Donghui Long

**Affiliations:** 1 State Key Laboratory of Chemical Engineering, East China University of Science and Technology, Shanghai 200237, China

## Abstract

We demonstrate a sustainable and efficient approach to produce high performance sulfur/carbon composite cathodes via a bottom-up catalytic approach. The selective oxidation of H_2_S by a nitrogen-enriched mesoporous carbon catalyst can produce elemental sulfur as a by-product which *in-situ* deposit onto the carbon framework. Due to the metal-free catalytic characteristic and high catalytic selectivity, the resulting sulfur/carbon composites have almost no impurities that thus can be used as cathode materials with compromising battery performance. The layer-by-layer sulfur deposition allows atomic sulfur binding strongly with carbon framework, providing efficient immobilization of sulfur. The nitrogen atoms doped on the carbon framework can increase the surface interactions with polysulfides, leading to the improvement in the trapping of polysulfides. Thus, the composites exhibit a reversible capacity of 939 mAh g^−1^ after 100 cycles at 0.2 C and an excellent rate capability of 527 mAh g^−1^ at 5 C after 70 cycles.

Lithium-sulfur (Li-S) batteries are expected to be the next-generation energy storage system to provide much higher energy density^1^,^2^,^3^. Sulfur cathodes offer an order of magnitude higher capacity at an operating voltage of 2.1 V^4^. While the high capacity can significantly enhance the energy density of the batteries, the lower operating voltage can also offer better safety^5^,^6^. However, there are still several major issures for sulfur cathodes inherent in the cell chemistry: (i) poor cycle life and high self-discharge due to the dissolution of the polysulfide intermediates, and (ii) low utilization and limited rate capability of sulfur cathodes due to the insulating nature of sulfur and the discharge product^7^,^8^.

In response to these challenges, current state-of-the-art strategy is coupling sulfur phases with the conductive materials^9^,^10^. Via melt diffusion or wet impregnation, sulfur could be easily placed into various carbon hosts, such as carbon blacks^11^, activated carbons^12^,^13^, carbon nanotubes^14^, graphene^15^,^16^,^17^ and mesoporous carbons^18^,^19^. In this way, the conductive carbon framework constrains the sulfur within its channels and generates essential electrical contact. And the nanopores of the carbon can accommodate sulfur volumetric expansion. Meanwhile, kinetic inhibition to diffusion within the confined channels and the sorption properties of the carbon both aid in trapping the intermediate polysulfides. As a result, the effective utilization of sulfur and the cycling stability are certainly improved. Furthermore, the characteristics of the carbon hosts, such as pore size^20^, porosity^21^, surface chemistry^22^,^23^, morphology^24^,^25^ and polymer coating^26^, are also critically important to ensure efficient use of the sulfur. Although improvements are obtained, the capacity fading with cycling is still an intrinsic problem for the sulfur/carbon composites. It is inevitable that with the top-down approaches, where sulfur is melt-or wet-impregnated into pre-synthesized carbon hosts, the sulfur aggregate and pore blockage are still major issues that result in the dissoultion of polysulfides in the sulfur/carbon composites. This puts forward higher challenges for the carbon hosts, which call for an even stronger role in effectively trapping polysulfides. Beside the carbon, it also needs an efficient and scalable approach to prepare the advanced sulfur/carbon composites, allowing the sulfur to be evenly distributed on the surface of carbon framework.

Herein, we report a sustainable, scalable and efficient approach to produce high performance sulfur/carbon composite cathodes via a bottom-up catalytic oxidation approach. We recently found that the nitrogen-enriched mesoporous carbons (NMCs) can act as metal-free catalysts in their own right to directly oxidize H_2_S to elemental sulfur at the room temperature via the reaction H_2_S + 1/2O_2_ → S + H_2_O^27^. Keys to the reaction lie in the high-content nitrogen atoms incorporated into the mesoporous carbon framework that can trigger the catalytic activity of the carbon towards H_2_S oxidation^28^. Unparalleled sulfur capacity was achieved for these NMCs, making them on the verge of being exploited in large-scale industrial H_2_S removal. Moreover, metal-free catalytic characteristic and high catalytic selectivity could exclude the presence of impurities, thus allowing these by-product NMC/S composites for further applications. While evaluated as cathode materials, these NMC/S composites show a high reversible capacity together with the excellent cycle stability and rate performance. Instead of making sulfur composites cost, the unique NMC/S composites could capture the inherent economic value of by-product themselves from H_2_S removal industry. And moreover, their intrinsic structure benefits could provide new implications for designing advanced sulfur/carbon composites for Li-S batteries. Firstly, relative to the conventional top-down impregnation, bottom-up catalytic synthesis of NMC/S composites allows sulfur atoms being layer-by-layer deposited on the carbon framework. In this way, the NMC/S composites where sulfur atoms is located very close to the conductive carbon, provide efficient immobilization of sulfur and the enhancement of electronic contacts with sulfur, as a direct consequence to improved electrochemical properties. Secondly, a large amount of nitrogen atoms doped on the carbon framework can significantly enhance the electronic conductivity of the carbon. And the basic nitrogen functionalities may increase the surface interactions with polysulfide anions, leading to the improvement in the trapping of polysulfides. These structural and surface chemistry features are expected to work co-operatively and lead to the NMC/S composites with high electrochemical performances.

## Results

**Figure 1:**
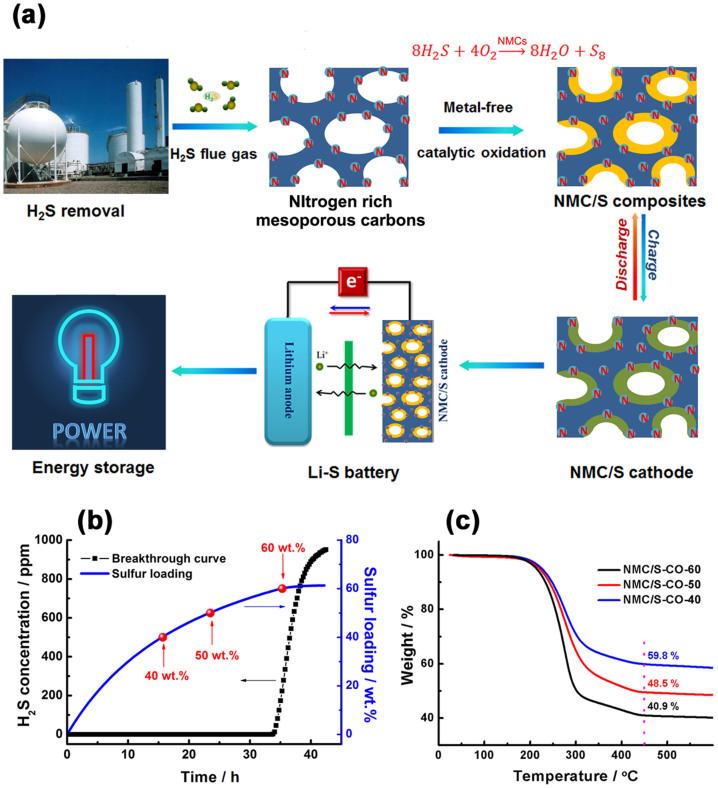
(a) The concept of waste to wealth that the metal-free H_2_S desulfurizer into unique sulfur/carbon cathodes for energy storage. (b) H_2_S breakthrough curves over the NMCs and the theoretical sulfur loadings calculated from H_2_S breakthrough curves. (c) Thermogravimetric analysis of NMC/S-CO series.

As we previously demonstated, it was possible to transform traditional mesoporous carbons into a superior metal-free catalyst for low-temperature H_2_S removal via doping a high concentration of nitrogen atoms into the carbon^27^. The nitrogen could introduce basic/catalytic sites to the carbon, which can increase surface polarity and enhance electron-donor properties of the carbon matrix, and in turn, increase the chemical reactivity for acid-base or redox chemistry. During the catalytic reaction, the oxidation product in the form of elemental sulfur S_8_ can be *in-situ* deposited on the carbon framework of the NMCs, and the homogenous NMC/S composites are formed as a by-product. A central motivation of the present work was to enable the waste by-product into a high-value cathode material that still retained high electrochemical activity of elemental sulfur, as an illustration of waste to wealth shown in Figure 1a.

The NMCs used in this work were prepared by a templating approach using melamine, phenol and formaldehyde as the carbon precursors and commercial colloidal silica as hard templates, similar to our previous report^29^. The as-obtained NMCs have two characteristic features including developed mesoporous structure and high level of nitrogen doping. Typically, the NMCs have a BET surface area of 580 m^2^ g^−1^, a total pore volume of 2.4 cm^3^ g^−1^ and an average mesopore size of 12 nm. In addition, the NMCs have a high nitrogen content of 7.9 wt.% determined from elemental analysis or 19.1 at.% from XPS result. The variation of the values can be explained by the surface specificity of XPS measurements, suggesting the N atoms are apt to gather in the surface rather than the bulk of carbon framework. More detailed characterizations of the NMCs are summarized in Figure S1–S3 and Table S1.

The oxiation reaction was carried out in a packed-bed reactor under atmospheric pressure at a temperature of 30°C. As shown in Figure 1b, the theoretical sulfur content in the composites could be calculated according to the H_2_S breakthrough curve, which could be controlled by adjusting the reaction time. To facilitate the evaluation of electrochemical performance, here we prepared three NMC/S composites at the H_2_S duration time of 16 h, 24 h and 35.5 h, respectively. These composites are denoted as NMC/S-CO-40, NMC/S-CO-50 and NMC/S-CO-60, where 40, 50 and 60 represent the sulfur contents in the NMC/S composites respectively and the notation CO represents the catalytic oxidation method. The sulfur content of each sample is further confirmed by thermogravimetric analysis (TGA) in Figure 1c, which are 40.9 wt.%, 48.5 wt.% and 59.8 wt.%, respectively, in good agreement with their theoretical sulfur loadings.

**Figure 2:**
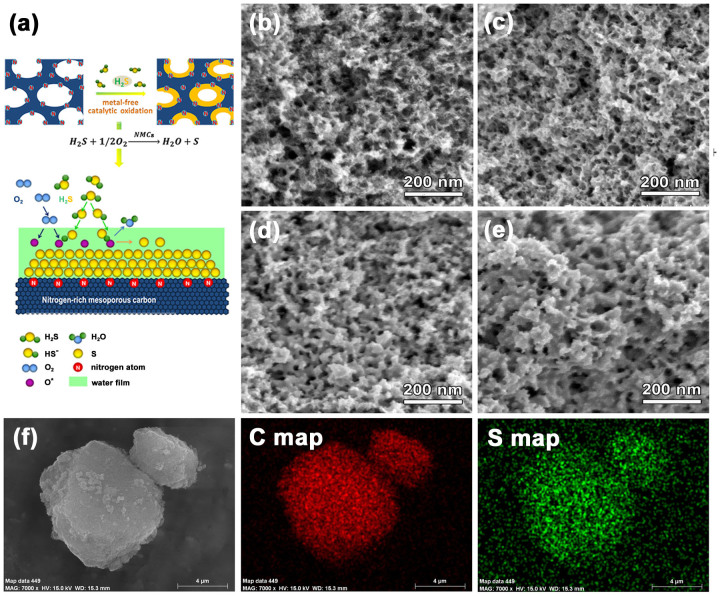
(a) Schematic diagram of the *in-situ* growth of atomic sulfur layer on the surface of NMCs framework. SEM images of fresh NMCs (b), NMC/S-CO-40 (c), NMC/S-CO-50 (d) and NMC/S-CO-60 (e). SEM mapping of NMC/S-CO-60 (f).

The sulfur atoms, originated from the catalytic oxidation of gaseous H_2_S, could be *in-situ* deposited on the surface of nitrogen-enriched carbon framework. This process illustrated in Figure 2a might be similar to the chemical vapor deposition process. H_2_S molecules diffuse into the pores and dissolve into the water film on the surface of the carbon framework. The presence of nitrogen groups as Lewis basic sites could increase the local basicity of the water film, which could facilitate the dissociation of H_2_S into HS^−^ ions (pk 6.89). Meanwhile, oxygen molecules diffuse into the pores and are preferentially adsorbed by the active sites, particularly those pyridinic nitrogen atoms at graphitic edge plane sites. The subsequent reaction between high concentration HS^−^ ions and adsorbed oxygen radicals leads to the formation of the elemental sulfur. And then the formed sulfur atoms are suggested to self-catalyze the H_2_S oxidation, allowing the layer-by-layer growth of atomic sulfur. This growth process could be revealed by SEM observation as shown in Figure 2b–e. After the oxidation reaction, the carbon framework is homogenously wrapped by the product sulfur. The thickness of sulfur layer gradually increases as the reaction progress, and no discernible sulfur agglomerations are found on the surface of NMCs. The SEM elemental mapping images (Figure 2f and Figure S4) and STEM elemental mapping images (Figure S5) show matched spatial distributions of S and C, indicating the uniform distribution of sulfur within the carbon matrix.

**Figure 3:**
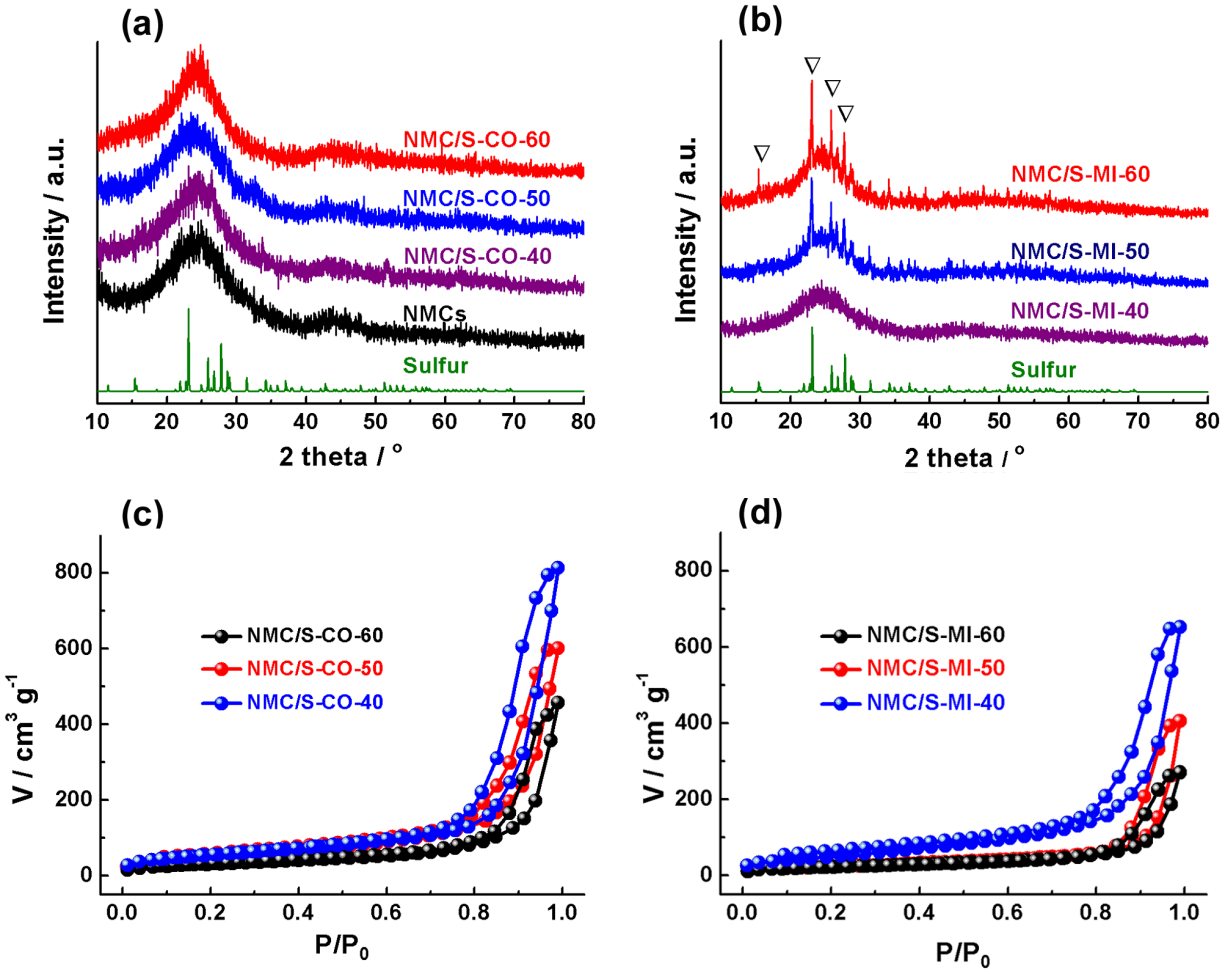
XRD patterns of NMC/S-CO series (a) and NMC/S-MI series (b). N_2_ adsorption-desorption isotherms of NMC/S-CO series (c) and NMC/S-MI series (d).

XRD analysis is conducted on NMCs and NMC/S composites, as shown in Figure 3a. There are almost no obvious differences between these samples, even a large amount of sulfur condensed within the mesopores. This result indicates that the product sulfur should be confined in the very internal parts of carbon matrix that cannot crystallize into large size. XPS analysis is employed to confirm the chemical nature of NMC/S composites. In the range of XPS sensitivity, only carbon, nitrogen, oxygen and sulfur are detected in the survey scans (Figure S6a), thus excluding the presence of other impurities. The XPS S_2p_ spectra reveals (Figure S6b) that the sulfur is in the dominated form of elemental sulfur consisting of the S_2p3/2_ and S_2p1/2_ components.

The bottom-up deposition of sulfur atoms should have significant advantages in terms of the sulfur dispersion, compared to the top-down impregnation which requires the bulk diffusion of sulfur phases. For the comparison, we prepared three NMC/S counterparts based on the classical melt impregnation^30^. The sulfur content of these samples (denoted as NMC/S-MI-40, NMC/S-MI-50 and NMC/S-MI-60, where the notation MI represents the melt impregnation method) are determined to be 40.7 wt.%, 51.0 wt.% and 60.3 wt.% sulfur, respectively, according to TGA results (Figure S7b). This could thus minimize the influence of the sulfur content, which could prompt us to focus on the effects of sulfur loading method on the properties of the resulting composites. Compared with the melt-impregnation sulfur, the catalytic-growth sulfur has lower molecular unit or smaller microcrystalline size. This could be confirmed by XRD that the melt-impregnation sulfur contains typical *Fddd* orthorhombic sulfur diffraction peaks (Figure 3b) that do not present for the catalytic-deposition sulfur. In addition, the melt-diffusion sulfur does not display the same homogenous coating of sulfur over the carbon framework as the catalytic-growth sulfur (Figure S8). Some mesopores are seemed to be blocked by the sulfur aggregates, owing to the random filling of sulfur during the melt diffusion.

Figure 3 c and d compare N_2_ adsorption-desorption isotherms of the catalytic-growth NMC/S composites and their melt-impregnation counterparts. After loading of the sulfur, the BET surface area and the pore volume of the NMCs composites decrease markedly for all the samples. However, in the case of the same sulfur loading, the NMC/S-CO series samples exhibit larger pore volume and higher surface area than the NMC/S-MI series (Table S2). Undoubtedly, the variation of these values is due to the intimate contact of sulfur with the carbon framework that creates extra porosity at the same sulfur loading. This point further confirms that the catalytic strategy could produce more uniform sulfur/carbon composites than the conventional impregnation.

The nitrogen doping can enhance conductivity of the carbon matrix and increase surface polarity with an improved weak chemisorption activity for molecules^31^,^32^. These inspire us to investigate the role of nitrogen doping on the electrochemical performance when the NMCs work as the hosts for the sulfur cathodes. To this end, nitrogen-free mesoporous carbons (MCs) are also synthesized by the colloidal silica nanocasting only using phenol and formaldehyde as the carbon precursors. The as-prepared MCs have very similar porous structure with the NMCs (Figure S2, Table S1). To facilitate the comparison, the sulfur composites based on MCs were also prepared under the same melt-impregnation conditions as those used for NMC/S-MI series. The resulting composites are denoted as MC/S-MI-40, MC/S-MI-50 and MC/S-MI-60 respectively, which are also characterized in detail (Figure S7c, Table S2).

**Figure 4:**
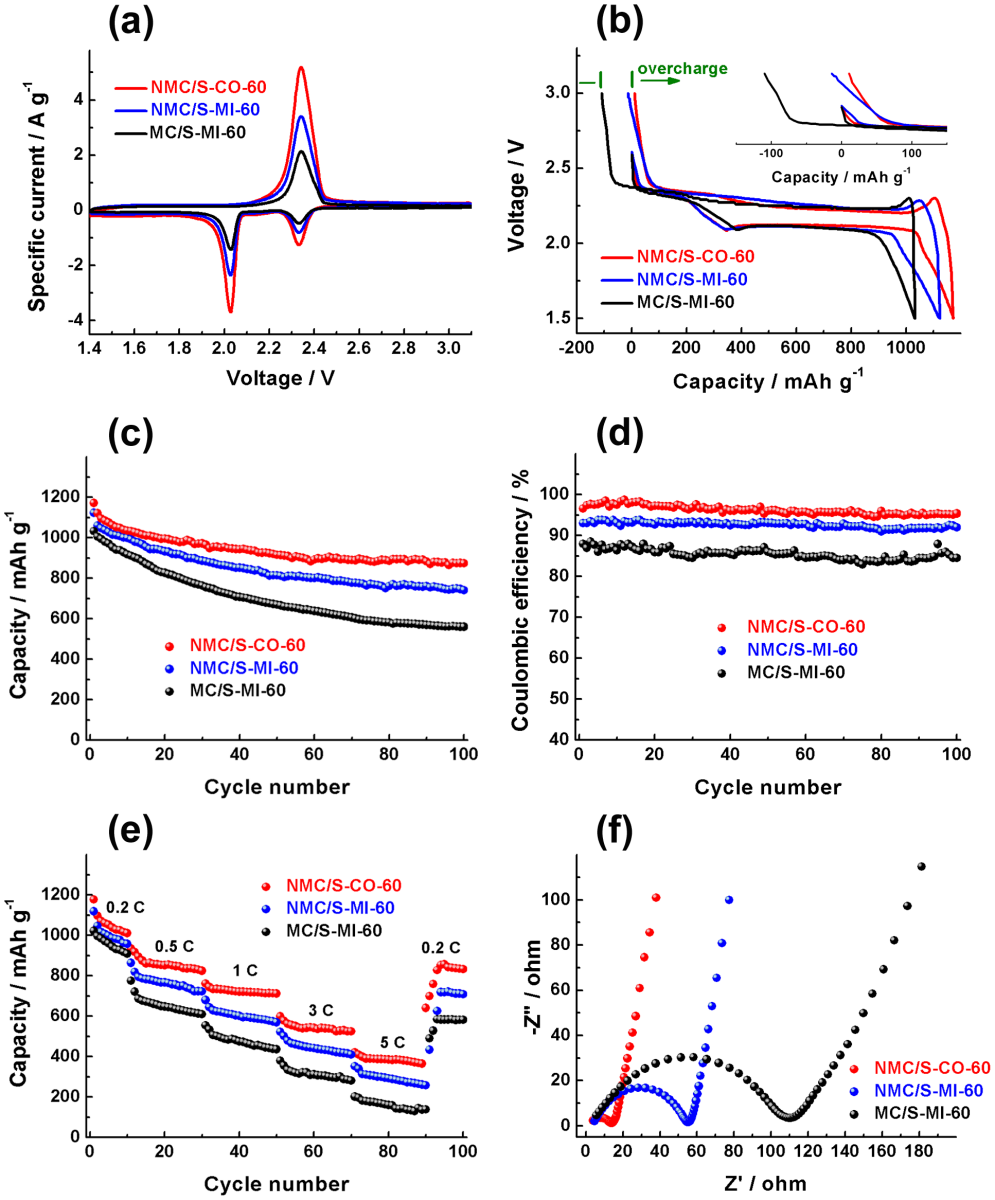
Electrochemical behaviors of NMC/S-CO-60, NMC/S-MI-60 and MC/S-MI-60: cyclic voltammograms at 0.2 mV s^−1^ (a); initial charge-discharge curves (b), cycle stability (c) and coulombic efficiency (d) at 0.2 C; rate capacity (e); EIS before 1st cycle (f). Inset in (b) shows overcharge capacity of NMC/S-CO-60, NMC/S-MI-60 and MC/S-MI-60. The C rate was based on the theoretical capacity of sulfur (1 C = 1675 mA g^−1^).

The electrochemical behaviors of three series of the composites (NMC/S-CO, NMC/S-MI and MC/S-MI) were systematically studied by cyclic voltammetry (CV), galvanostatic charge-discharge testing, and electrochemical impedance spectroscopy (EIS). We emphasize that it is important to have sulfur atoms deposited closely onto the nitrogen-doped carbon framework instead of melt-filling the bulky sulfur molecules. Here, three series of composites with 60 wt.% sulfur are exemplified as shown in Figure 4.

Figure 4a shows the CV of the composites with 60 wt.% sulfur scanned at 0.2 mV s^−1^. Two well-defined reduction peaks for all the composites exist distinctly, which are centred at 2.32 and 2.01 V corresponding to the two step conversion of high-order lithium polysulfides (e.g., Li_2_S_8_) to low-order lithium polysulfides (Li_2_S_x_, 4 ≤ x < 8) and lithium polysulfides to solid-state Li_2_S_2_/Li_2_S, respectively^33^,^34^. Except for the intensity difference of these peaks, there are no other obvious differences observed. Figure 4b shows the first discharge and charge profiles of the composites recorded at current density of 0.2 C. Two plateaus are observed for all samples in the discharge process, which are the typical characteristics of sulfur/carbon cathodes. The MC/S-MI-60 exhibits an obvious overcharge capacity of ca. 100 mAh g^−1^ (inset in Figure 4b), a typical signal of the polysulfide shuttle^35^. The results of the other MC/S-MI series, which are shown in Figure S12a–b, are also similar, suggesting only mesoporous carbon framework cannot entirely prevent the shuttle phenomenon. However, doping nitrogen atoms into MCs could greatly lower overcharge capacity. The basic nitrogen functionalities are proposed to serve as immobilizers to anchor polysulfide anions, which could thus retard shuttle of polysulfides^36^. Moreover, the shuttle phenomenon could be further suppressed by optimizing the sulfur dispersion state via the catalytic route. These results suggest that the uniform dispersion of sulfur and its close contact with nitrogen-doped carbon framework should effectively limit the diffusion of the polysulfide anions out from the mesopores into the electrolyte.

Figure 4c compares the cycle life of three series composites. The MC/S-MI-60 shows an impressive discharge capacity of 1033 mAh g^−1^ (at a rate of 0.2 C) in the first cycle, which drops to 560 mAh g^−1^ after 100 cycles. This is similar to that reported for other mesoporous carbon-based cathodes^34^,^37^,^38^,^39^. Nevertheless, the nitrogen doping could aid MCs to improve the cycling performance in a large degree. The retention rate of the capacity of the NMC/S-MI-60 significantly increases to 66%, with a first discharge capacity of 1123 mAh g^−1^ and 741 mAh g^−1^ after 100 cycles. Furthermore, the NMC/S-CO-60, a prototype of the waste by-product from H_2_S removal, exhibit much better cycling performance, delivering an initial discharge capacity of 1172 mAh g^−1^ and a reversible capacity of 874 mAh g^−1^ after 100 cycles. The origin for this enhanced cycling performance can be ascribed to a better immobilization of sulfur and polysulfide species by the NMCs, which maintain the structural integrity of the composites during the long-term cycling as observed by SEM images and EIS measurements (Figure S9–S10). Additionally, the coulombic efficiency (Figure 4d) can also improve by nitrogen doping from 88% to 94% and further increase to 97% by the catalytic approach, indicating very stable reversibility of the electrochemical reactions.

The rate capabilities of three series of composites at various rates are compared in Figure 4e. At the maximum discharge rate of 5 C (8.5 A g^−1^), the NMC/S-CO-60 delivers 420 mAh g^−1^, much higher than its melt-impregnation counterparts. The excellent stability of the NMC/S-CO-60 is also evidenced by the recovery of a capacity of 854 mAh g^−1^ at 0.2 C. The outstanding kinetic behavior should be attributed to the improved electronic and ionic transport at the cathode. This could be further confirmed by the EIS measurements (Figure 4f), which reveal that the NMC/S-CO series composites exhibit much lower charge-transfer resistances and diffusion impedances than the melt-impregnation samples. In addition, the EIS results of NMCs evaluated as electrode materials in the supercapacitors also suggest the significantly enhanced charge-tranfer kinetics caused by nitrogen doping (Figure S11). Conductivity measurements give further insights into the positive role of dispersion state and nitrogen doping on the electric conductivity of the composites. The conductivity of pristine MCs was found to be 0.09 S cm^−1^, which increase to 0.29 S cm^−1^ by nitrogen doping. After sulfur loading, the NMC/S-CO-60 exhibits a conductivity of 0.25 S cm^−1^, slightly higher than 0.20 S cm^−1^ of NMC/S-MI-60 and much higher than 0.06 S cm^−1^ of MC/S-MI-60. Such improved conductivity should be desirable and significant for faster electronic transport.

Similarly, the overall electrochemical properties of other NMC/S-CO series composites are also much better than their NMC/S-MI and MC/S-MI counterparts (Figure S12). Higher reversible capacity and better cycle stability are achieved for the NMC/S-CO-40, which exhibits a reversible capacity of 939 mAh g^−1^ after 100 cycles at 0.2 C and an excellent rate capability behavior of 527 mAh g^−1^ at 5 C after 70 cycles (Figure S13). Such excellent electrochemical performance should be among the best series of carbon-based sulfur cathode materials, which are definitely ascribed to their unique structure issued from the catalytic reaction approach.

## Discussion

For the conventional preparations of carbon-based sulfur cathodes, elemental sulfur is placed into the carbon species by using thermal evaporation, sulfur solution, etc., which cannot promise rapid charge transfer and prevent the shuttle phenomenon. In contrast, the metal-free catalytic oxidation of H_2_S over NMCs produces the atomic sulfur that could be bottom-up deposited on the surface of carbon framework, allowing its intimate interaction with the carbon framework. The interaction could provide higher interface area for electron transfer, and also anchor effectively the active materials. The empty interconnected channels can also preserve fast transport of ions and accommodate the large volumetric expansion of sulfur during cycling. Furthermore, the sulfur formed in the atomic level might be more capable of reversibly cleaving and reforming on reduction and oxidation in the molecular skeleton. Apart from the structural characteristic of the composites, the presence of a large amount of nitrogen atoms on the surface of carbon framework can enhance the electronic conductivity of the carbon. In addition, basic nitrogen functionalities may trigger strong adsorbing ability to anchor the polysulfides anions, aiding the carbon to slow down the shuttle problem. Thus, the unique NMC/S-CO composites could indeed improve the effective utilization of sulfur for high power Li-S batteries. The low cost and amenability of large-scale production of the sulfur composites from H_2_S removal industry are also distinctive advantages compared with the conventional preparation methodes.

In summary, we demonstrate a bottom-up approach to build nitrogen-enriched mesoporous carbon/sulfur nanocomposites from metal-free catalytic oxidation of H_2_S for superior Li-S cathodes. The synergetic effect of nitrogen doping and mesoporous structure could fulfill the catalytic reaction of H_2_S oxidation over the carbons, which produces the atomic sulfur that *in-situ* deposits onto the carbon framework, forming the unique NMC/S composites. The as-prepared NMC/S composites with outstanding structural features can help solve the interrelated challenges of poor ionic and electronic transport and polysulfide dissolution in the sulfur cathodes, and thus exhibit good reversibility, excellent capacity stability, and rate capability of up to 5 C. These encouraging results show the sulfur dispersion state from the catalytic strategy and nitrogen doping are good strategies for the high performance sulfur/carbon cathodes. We believe that these strategies can be introduce into other carbon materials with proper porosity, surface chemistry and nano-morphology, in order to homogenously disperse sulfur, thereby improve the electrochemical properties for the Li-S cathodes.

## Methods

### Synthesis

For the synthesis of nitrogen-rich mesoporous carbons (NMCs), 18.35 g phenol (195 mmol) and 31.65 g formaldehyde (37 wt.%, 390 mmol) were dissolved in 250 ml of 0.2 M NaOH solution (50 mmol). The mixture was stirred at 70°C for 40 min. Then, 24.6 g melamine (195 mmol) and 47.5 g formaldehyde (535 mmol) were added to the above solution to react for 30 min with consecutive agitation until the solution became clear. Next, 250 g Ludox HS-30 sol (30 wt.% SiO_2_) was added to the above solution under stirring. The weight ratio of polymer precursor to colloidal silica was 1:1. The mixture was transferred to a large sealed bottle and heated at 80°C for 3 days. The obtained hydrogels were directly dried in an ambient condition, followed by the carbonization at 800°C for 3 h in a nitrogen flow. The NMCs were obtained by dissolution of the silica nanoparticles in 2 M NaOH solution at 80°C, isolated by filtration, washed several times with distilled water and ethanol, and dried at 100°C.

The NMC/S-CO composites were prepared via a selective catalytic oxidation of H_2_S over NMCs. The reaction was carried out in a packed-bed reactor under atmospheric pressure. An influent concentration of 1000 ppmv of H_2_S in simulated mixture (N_2_ 99%, O_2_ 1%, flow rate 150 ml min^−1^, weight hourly space velocity of 5.5 h^−1^, operating temperature 30°C and relative humidity 80%) was used. For direct electrochemical applications, the NMCs were ball-milled into superfine powder and then packed with quartz sand in a glass reactor. According to the breakthrough curve, the NMC/S composites with sulfur content of 40 wt.%, 50 wt.% and 60 wt.% were obtained by terminate the reaction at the duration time of 16 h, 24 h and 35.5 h, respectively. The as-obtained products were immersed in the distilled water for overnight to remove trace H_2_SO_4_ on the surface of sulfur layers, and then dried in a vacuum oven at 60°C for 24 h.

For comparison, the NMC/S-MI composites were also prepared following a conventional melt-diffusion strategy^30^. The sublimed sulfur and as-prepared NMCs with various weight ratios were mixed homogeneously. The mixture was degassed in a vessel and then sealed under vacuum. The melt infiltration was further carried out in the vacuum-sealed vessel at 155°C for 10 h. Then the temperature was increased to 300°C for 2 h to vaporize the superfluous sulfur on the outer surface of carbon diffusing entirely into the pores.

The nitrogen-free mesoporous carbons (MCs) were also prepared by the above nanocasting method, only using phenol and formaldehyde as the carbon precursors. To match the porosity of the NMCs, the MCs were prepared at a weight ratio of polymer precursor to colloidal silica of 1:1.3. The sulfur composites based on MCs were prepared under the same melt-impregnation conditions as those used for NMC/S-MI series.

### Characterization

Elemental analysis was carried out using Elemental Vario EL III. The carbon (C), hydrogen (H), and nitrogen (N) contents of the carbons were determined directly using the thermal conductivity detector. The surface chemistry of the samples was analysed using an Axis Ultra DLD X-ray photoelectron spectroscopy. The thermogravimetric analysis (TA Instrument Q600 Analyser) of samples was carried out at a nitrogen flow rate of 100 ml min^−1^. The X-ray diffraction (XRD) patterns were acquired on a Rigaku D/max 2550 diffractometer operating at 40 KV and 20 mA using Cu Kα radiation (λ = 1.5406 Å). The morphologies of samples were observed under scanning electron microscopy (SEM, JEOL 7100F) and transmission electron microscopy (TEM, JEOL 2100F). The SEM mapping and energy dispersive X-ray spectroscopy (EDX) analysis were performed under scanning electron microscopy (SEM, FEI Q-300). The scanning transmission electronic microscope (STEM) was conducted on a Tecnai G2 F30. Nitrogen adsorption/desorption isotherms were measured at 77 K with a Quadrasorb SI analyser. Powder electrical resistivity measurements (FZ-2010) were carried out at room temperature using the four-contact method. The samples were filled in a teflon cylinder with inner diameter of 16 mm, and two stainless-steel plungers were used to deliver 4 MPa pressure through a hydraulic press device. The current and voltage through the two stainless-steel plungers were recorded using two keithley 2000 digital multimeters. The electrical conductivity was obtained based on the powder electrical resistivity.

### Electrochemical tests

The C/S materials were slurry-cast onto an aluminium current collector. Typically, 80 wt.% of C/S composite, 10 wt.% carbon black (Super P Conductive Carbon Black) and 10 wt.% PVDF were mixed with N-methyl-2-pyrrolidone (NMP). The slurries were coated on aluminium current collectors and dried at 60°C overnight. Electrochemical tests of these electrode materials were performed using coin cells with the sulfur composite cathode and lithium metal as the counter electrode. The electrolyte was 1 M bis(trifluoromethane) sulfonimide lithium salt (LiTFSI) dissolved in a mixture of 1.3-dioxolane (DOL) and dimethoxymethane (DME) (1:1 by volume). The separator was a microporous membrane (Celgard 2400). The cell was assembled in an argon filled glove box. The galvanostatic charge-discharge test and cyclic voltammetry measurements (CV) were conducted using an Arbin battery cycler (Arbin, BT2000, USA). All capacity values were calculated on the basis of sulfur mass. Electrochemical impedance spectroscopy (EIS) was performed with an electrochemical working station PCI4/300 (Gamry Instrument, Warminster, PA, USA). The sinusoidal excitation voltage applied to the coin cells was 5 mV, with frequency range from 100 kHz to 0.01 Hz. All the electrochemical tests were performed at 25°C.

## Author Contributions

F.G.S. and D.H.L. designed the experiments. F.G.S., H.C.C. and J.T.W. performed the experiments and prepared figures 1–4. J.T.W., W.M.Q. and L.C.L. involved in the scientific discussions. F.G.S. and D.H.L. co-wrote the main manuscript text paper. All authors reviewed the manuscript.

## Supplementary Material

Supplementary Information Supporting Inforamtion
