# Supplementary material for: Bottom-Up Catalytic Approach towards Nitrogen-Enriched Mesoporous Carbons/Sulfur Composites for Superior Li-S Cathodes
Source: Sci Rep. 2013 Oct 2;3:2823. doi: 10.1038/srep02823 (PMC3788363; doi:10.1038/srep02823)
Supplement: Supplementary Information — Supporting Inforamtion [file srep02823-s1.doc]

**Supplementary information**

**Bottom-Up Catalytic Approach towards Nitrogen-Enriched Mesoporous Carbons/Sulfur Composites for Superior Li-S Cathodes**

*Fugen Sun, Jitong Wang, Huichao Chen, Wenming Qiao, Licheng Ling & Donghui Long^*^*

*State Key Laboratory of Chemical Engineering, East China University of Science and Technology, Shanghai 200237, China.*

*To whom correspondence should be addressed. E-mail: [longdh@mail.ecust.edu.cn](mailto:longdh@mail.ecust.edu.cn);

**This PDF file includes:**

Experimental details

Table S1 to S2

Figure S1 to S13

**Content:**

**Table:**

**Table S1*.*** Chemical nature of the NMCs and MCs

**Table S2.** Pore structure parameters of the MCs and NMCs before and after sulfur loading

**Figure**:

**Figure S1.** XPS results of NMCs and MCs

**Figure S2.** N_2_ adsorption-desorption isotherms (a) and pore size distribution (b) of NMCs and MCs.

***Figure S3.*** Representative TEM image of NMCs.

***Figure S4.*** EDX spectrum of NMC/S-CO-60.

***Figure S5.*** STEM image of NMC/S-CO-60 (a), and the corresponding mixed elemental (C and S) mapping image (b), C mapping image (c) and S mapping image (d).

***Figure S6.*** XPS survey spectra (a) and S_2p_ spectra (b) of MC/S-MI-60, NMC/S-MI-60 and NMC/S-CO-60.

***Figure S7.*** Thermogravimetric analysis (TGA) curves of NMC/S-CO series, NMC/S-MI series and MC/S-MI series samples..

***Figure S8.*** SEM images of NMC/S-CO -60 (a) and NMC/S-MI-60 (b) samples.

***Figure S9.*** SEM images of MC/S-MI-60, NMC/S-MI-60 and NMC/S-CO-60 cathode before 1st cycle and after 20th cycle.

***Figure S10.*** EIS of NMC/S-CO-60, NMC/S-MI-60 and MC/S-MI-60 cathode before 1st cycle and after 20th cycle.

***Figure S11.*** EIS profiles of the MCs and NMCs evaluated as electrode materials in the supercapacitors.

***Figure S12.*** Initial charge-discharge curves, cycle capacity and EIS before 1st cycle of NMC/S series with sulfur loadings of 40 wt.% and 50 wt.%.

***Figure S13.*** Rate performance of NMC/S-CO series samples.

***Table S1.*** Chemical nature of the NMCs and MCs

| Samples | Element analysis | | XPS | | | | | S_BET_^[d]^ | V_T_^[e]^ |
| --- | --- | --- | --- | --- | --- | --- | --- | --- | --- |
|  | N | N/C | N | N/C | pyridinic N^[a]^ | pyrrolic N^[b]^ | graphitic N^[c]^ |  |  |
|  | wt.% | at./at. | at.% | at./at. | % | % | % | m^2^ g^-1^ | cm^3^ g^-1^ |
| MCs | - | - | - | - | - | - | - | 690 | 2.2 |
| NMCs | 7.9 | 0.08 | 19.1 | 0.3 | 56.3 | 31.6 | 12.1 | 580 | 2.4 |

[a] the relative concentration of pyridinic N (398.5±0.3 eV); [b] the relative concentration of pyrrolic N (400.5±0.3 eV); [c] the relative concentration of graphitic N (401.6±0.3 eV); [d] BET specific surface area; [e] total pore volume (P/P0= 0.985).

In our synthesis approach, the incorporation of high-nitrogen-content melamine into phenolic precursors can transfer nitrogen atoms into carbon framework under pyrolysis conditions. The N/C mole ratio of NMCs is 0.3 determined from high-resolution XPS, three times higher than the value obtained from elemental analysis. The variation of the values can be explained by the surface specificity of XPS measurements, suggesting the N atoms are apt to gather in the surface rather than the bulk of carbon framework.

**Table S2.** Pore structure parameters of the MCs and NMCs before and after sulfur loading

| Sample | S_BET_  m^2^ g^-1^ | S_mic_  m^2^ g^-1^ | V_t_  cm^3^ g^-1^ | V_mic_  cm^3^ g^-1^ |
| --- | --- | --- | --- | --- |
| NMC | 580 | 139 | 2.4 | 0.1 |
| MC | 690 | 291 | 2.2 | 0.2 |
| MC/S-MI-40 | 163 | 0 | 0.9 | 0 |
| MC/S-MI-50 | 81 | 0 | 0.6 | 0 |
| MC/S-MI-60 | 50 | 0 | 0.4 | 0 |
| NMC/S-MI-40 | 195 | 0 | 1.0 | 0 |
| NMC/S-MI-50 | 92 | 0 | 0.6 | 0 |
| NMC/S-MI-60 | 82 | 0 | 0.4 | 0 |
| NMC/S-CO-40 | 196 | 0 | 1.3 | 0 |
| NMC/S-CO-50 | 179 | 0 | 0.9 | 0 |
| NMC/S-CO-60 | 111 | 0 | 0.7 | 0 |


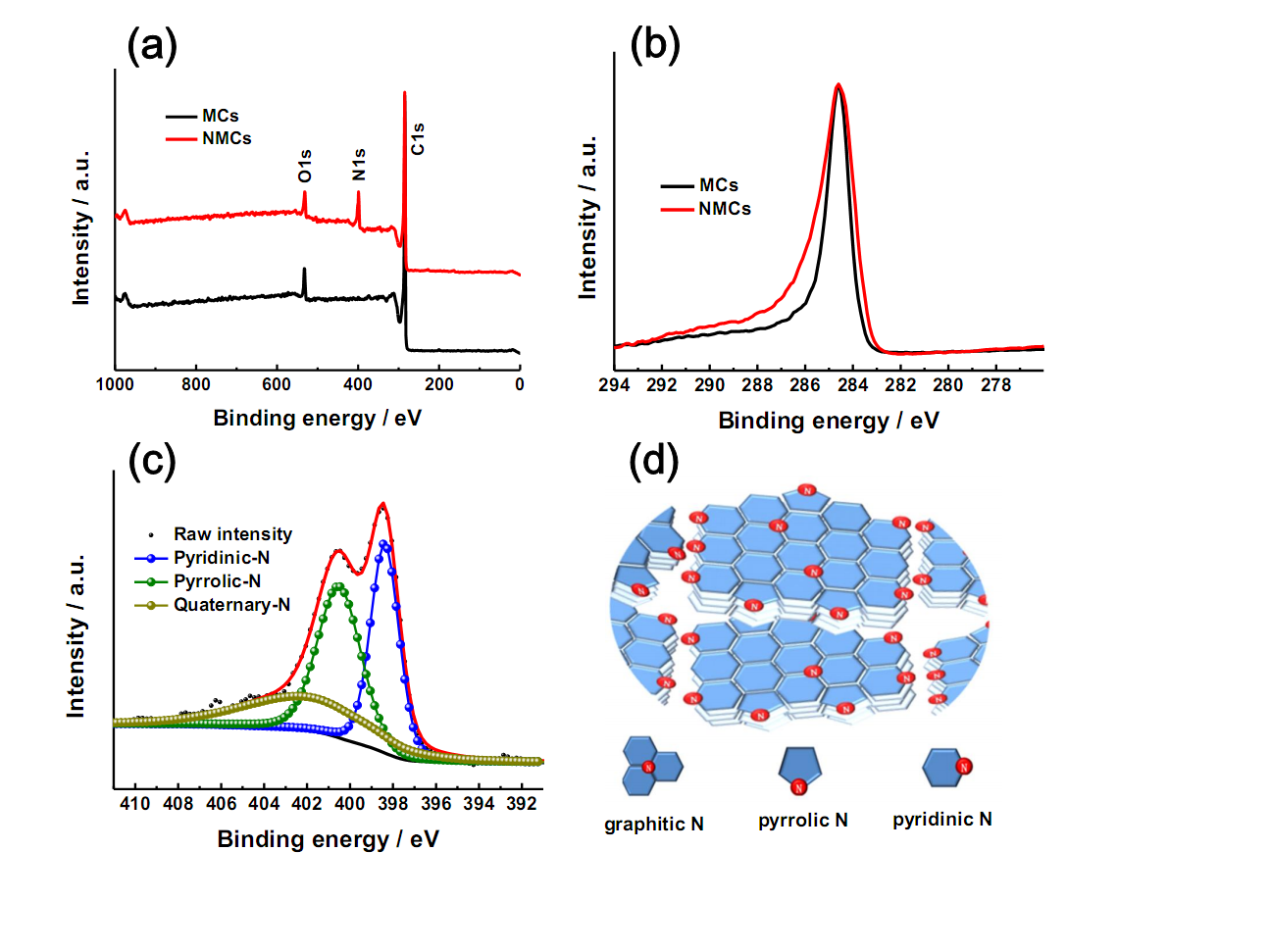


***Figure S1.*** (a) XPS survey spectra of NMCs and MCs; (b) high-resolution XPS C_1s_ spectra of NMCs and MCs; (c) high-resolution XPS N_1s_ spectra of NMCs; (d) schematic model of three nitrogen species and possible distribution in carbon framework of the NMCs. A strong N_1s_ peak is observed for NMCs, while no obvious peak corresponding to nitrogen is found for MCs. The C_1s_ peak of NMCs becomes wider than that of MCs, which might be caused by nitrogen doping. The N_1s_ spectra were curve-fitted into three peaks with binding energies of 398.5, 400.5 and 401.6 eV corresponding to pyridinic N (N1), pyrrolic N (N2), and graphitic N (N3), respectively.


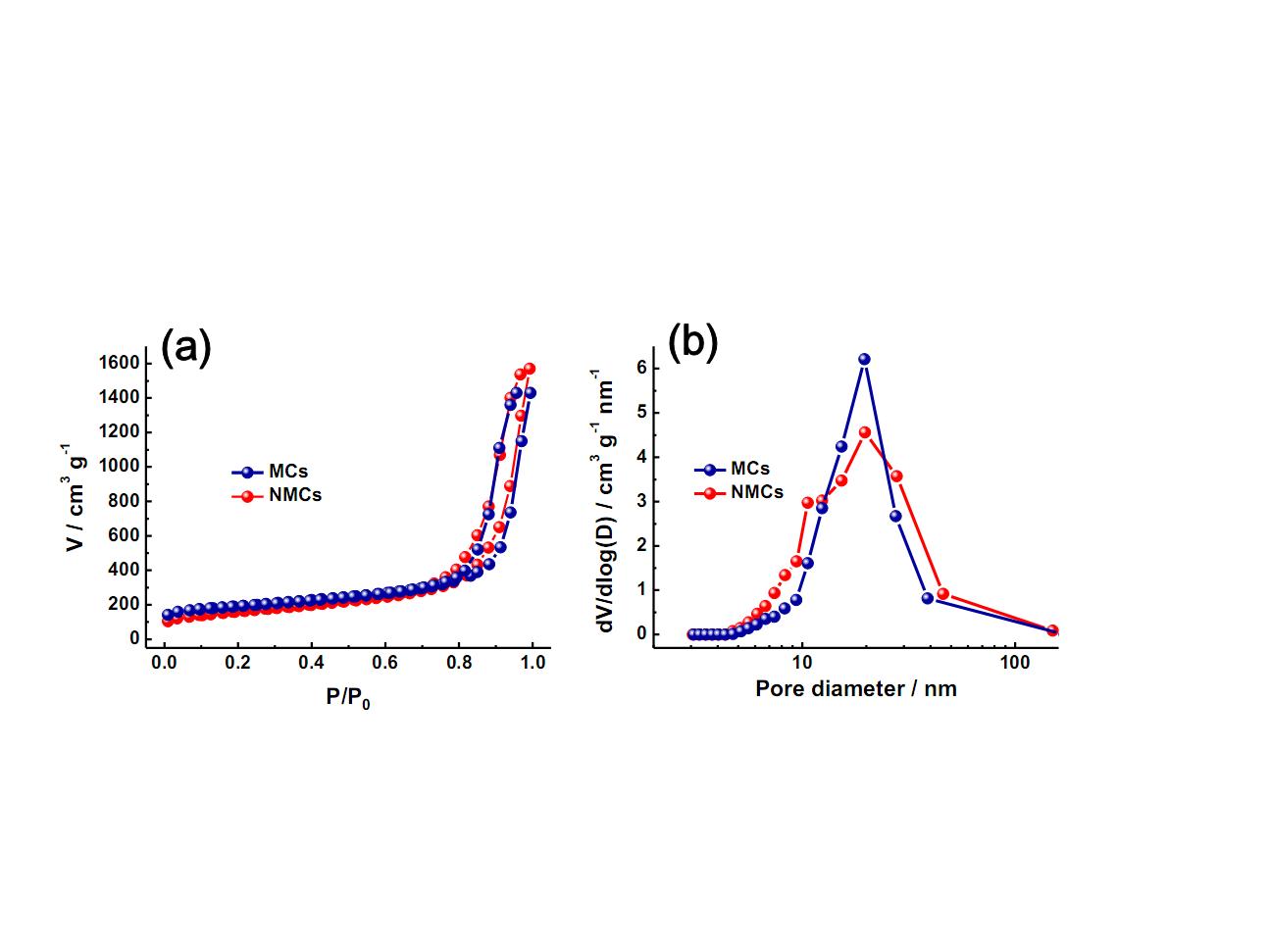


***Figure S2.*** N_2_ adsorption-desorption isotherms (a) and pore size distribution (b) of NMCs and MCs. The NMCs and MCs exhibit very similar isotherm type between type II and IV following the IUPAC classification, indicating that the NMCs and MCs are typically mesoporous carbon materials. The similar porosity stuctures of NMCs and MCs could minimize the influence of the porosity on the electrichemical performance, and prompt us to focus on the effect of the nitrogen doping on the performance of the carbon/sulfur cathodes.


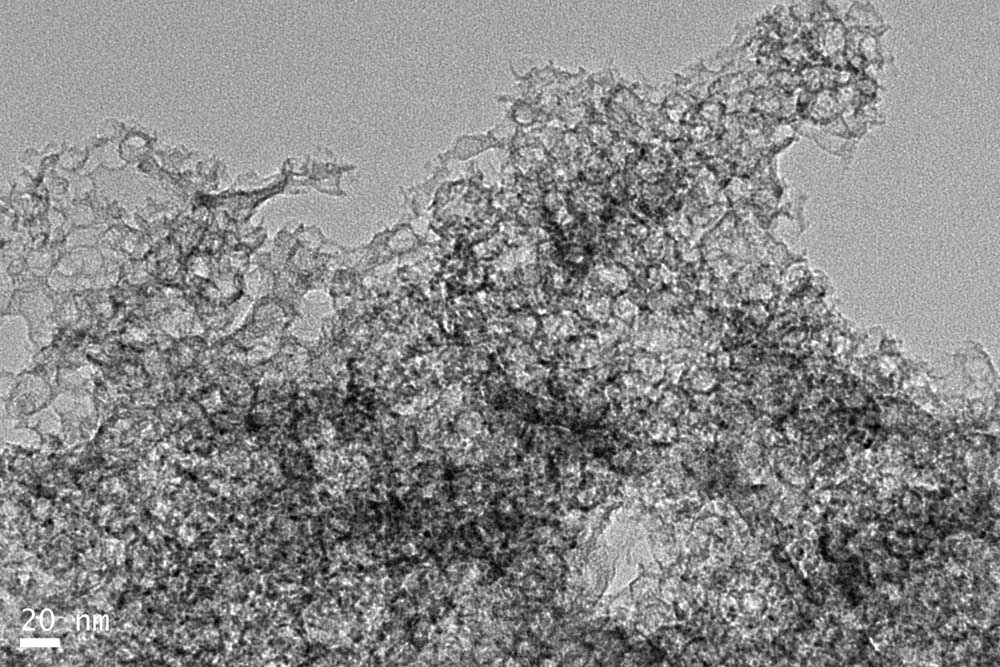


***Figure S3.*** Representative TEM image of NMCs. The pore size and connectivity of those NMCs exactly reflect the geometric properties of the original template. The well-developed uniform interconnected spherical mesopores can be regarded as a direct image, or more appropriately, a negative image of the structure of the colloidal silica particles.


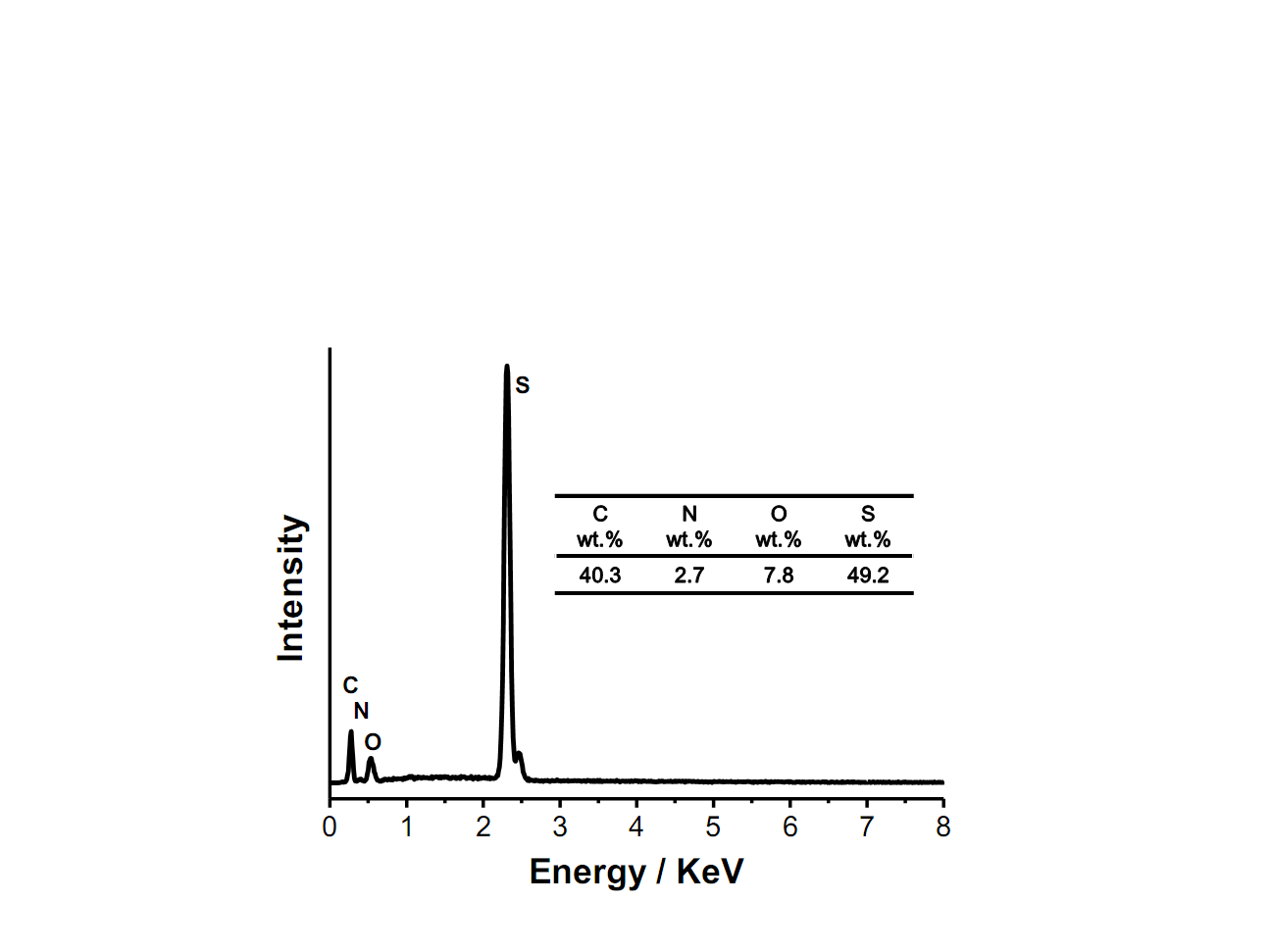


***Figure S4.*** EDX spectrum of NMC/S-CO-60. The strong intensity of sulfur signal in the EDX spectrum should indicate the presence of large amounts of sulfur in the NMC/S-CO-60 composites. Otherwise, the sulfur content of ca. 49.2 wt.% determined from EDX analysis is slightly lower than the real sulfur content of 60 wt.%. This should be due to the semi-quantitation specificity of EDX measurement and the unavoidable sublimation of sulfur in the high vacuum environment during the measurement.


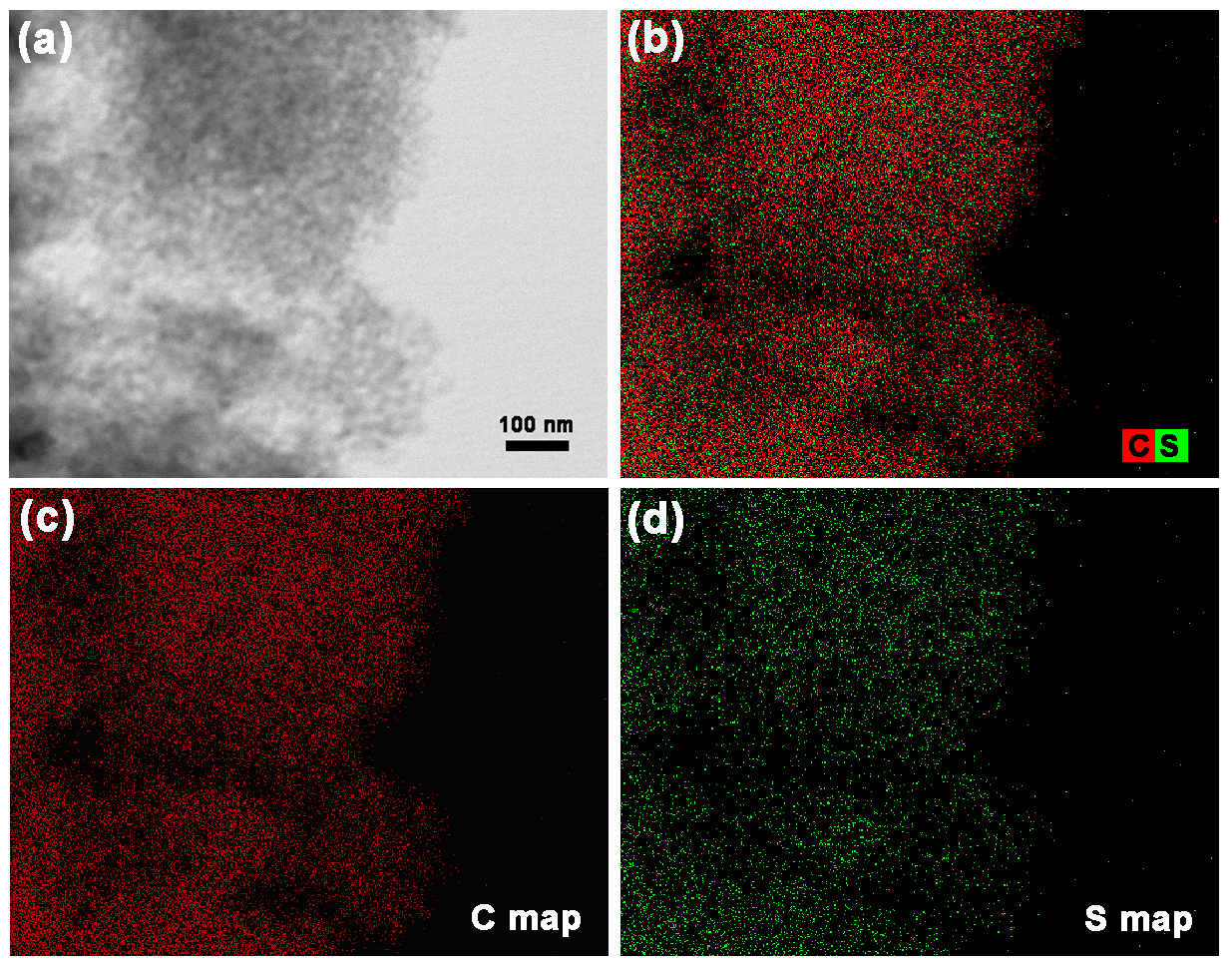


***Figure S5.*** STEM image of NMC/S-CO-60 (a), and the corresponding mixed elemental mapping image (b), C mapping image (c) and S mapping image (d). The STEM elemental mapping images also show matched spatial distributions of S and C, further confirming the uniform distribution of sulfur within the carbon matrix.


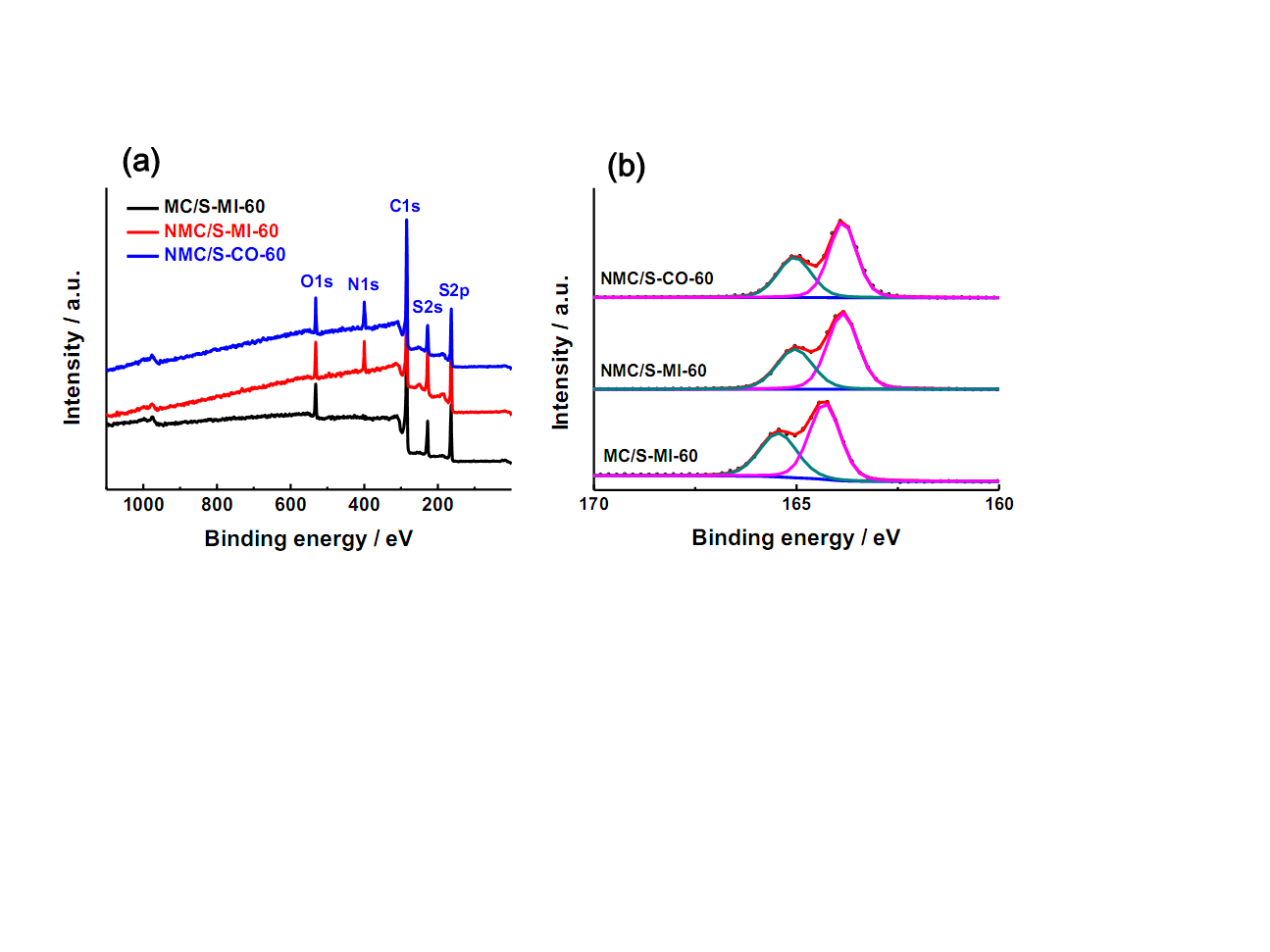


***Figure S6.*** XPS survey spectra (a) and S_2p_ spectra (b) of MC/S-MI-60, NMC/S-MI-60 and NMC/S-CO-60. In the range of XPS sensitivity, only carbon, nitrogen, oxygen and sulfur are detected in the survey scans thus excluding the presence of any other impurities. The high resolution S_2p_ signal of NMC/S-CO-60 can be curve-fitted into two peaks with the binding energies of 165.0 eV and 163.8 eV, corresponding to elemental sulfur present in the NMCs framework. No other peak of oxidated sulfur forms is observed.


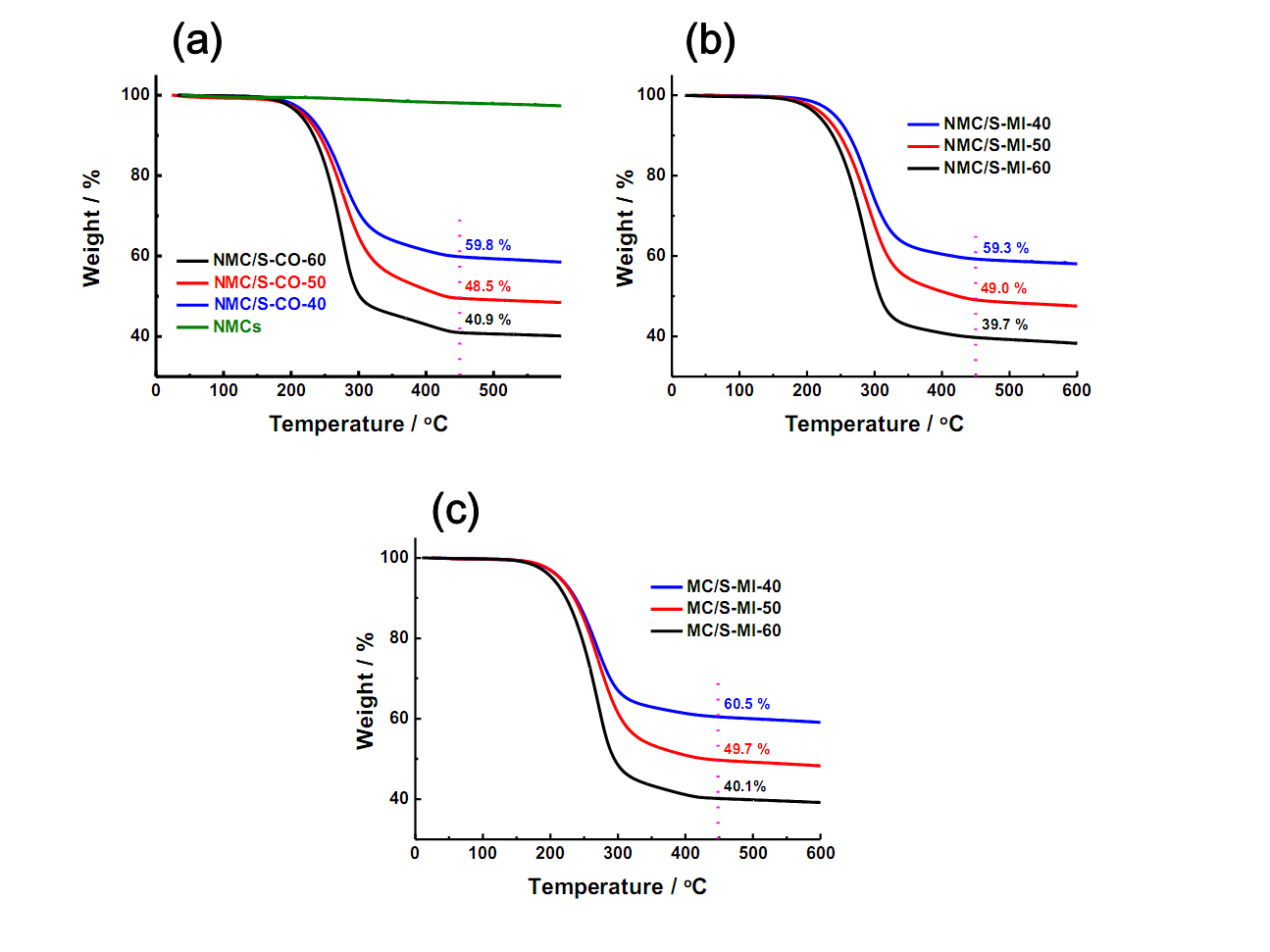


***Figure S7.*** Thermogravimetric analysis (TGA) curves of NMC/S-CO series samples (a), NMC/S-MI series samples (b) and MC/S-MI series samples (c). To facilitate comparison, the sulfur contents in each series are carefully controlled in same level. This could thus minimize the influence of the sulfur content, which could prompt us to focus on the effect of sulfur loading method on the electrochemical performance of the resulting composites.


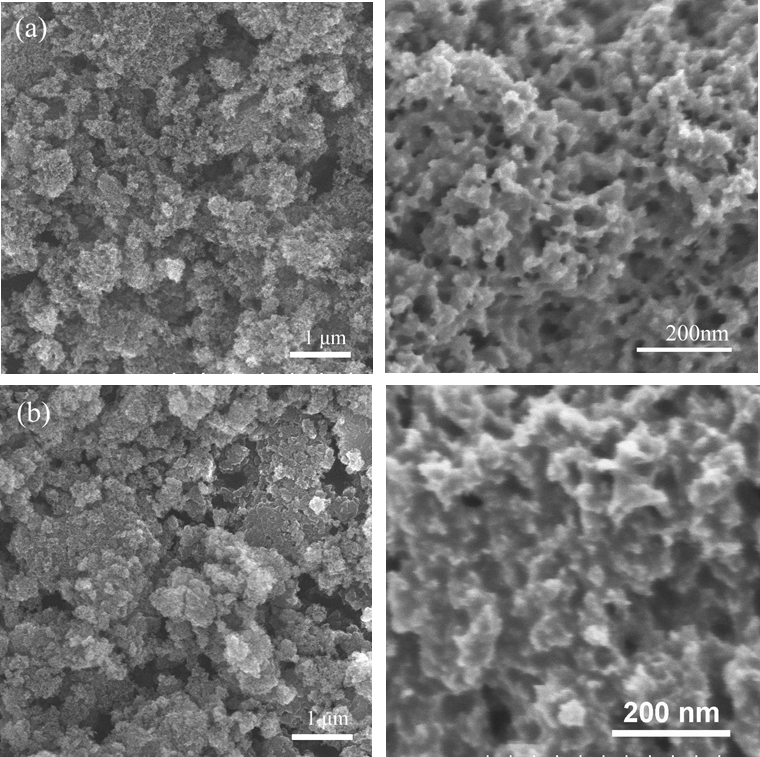


***Figure S8.*** SEM images of NMC/S-CO-60 (a) and NMC/S-MI-60 (b) samples. The melt-diffusion sulfur does not display the same homogenous coating of sulfur over the carbon framework as the catalytic-growth sulfur. Some mesopores are seemed to be blocked by the sulfur aggregates, owing to the random filling of sulfur during the melt diffusion.


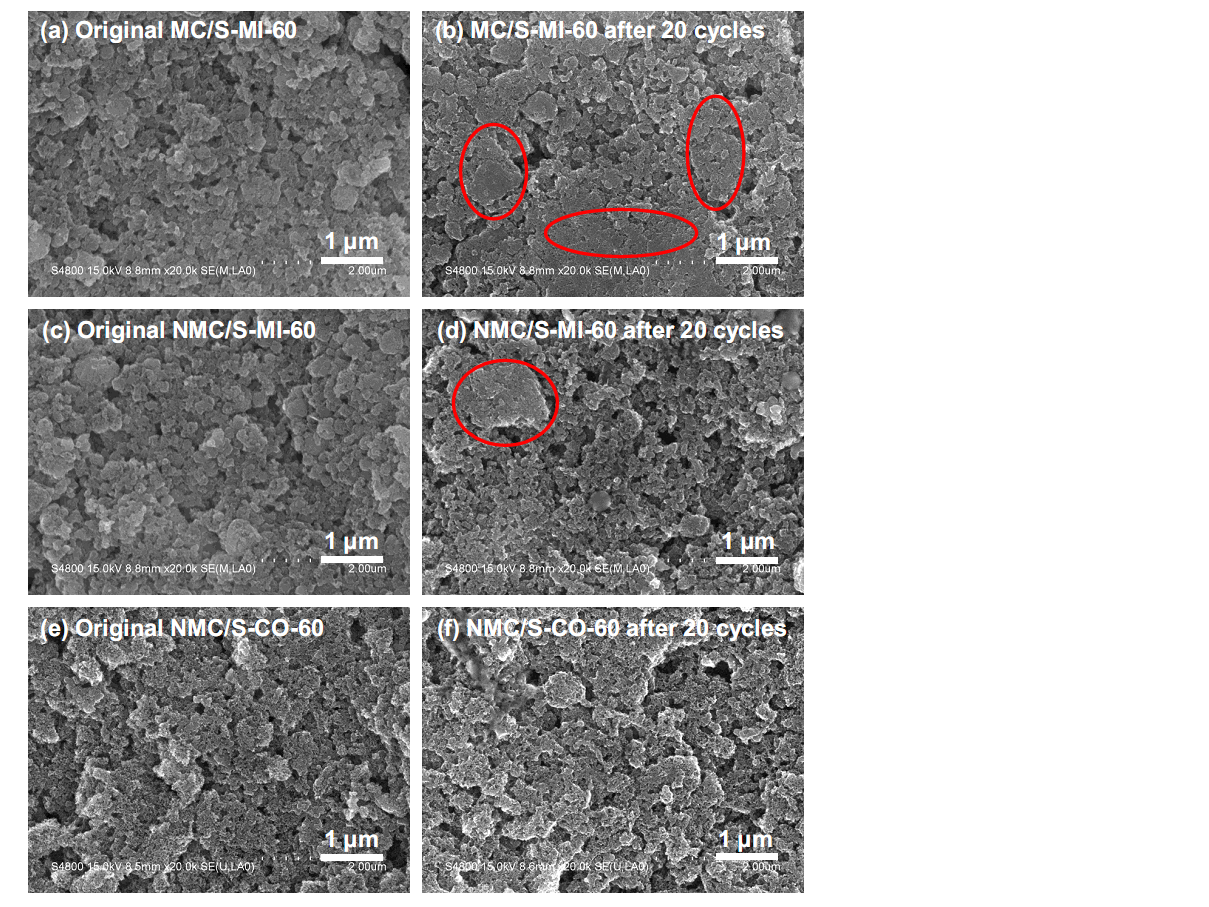


***Figure S9.*** SEM images of MC/S-MI-60, NMC/S-MI-60 and NMC/S-CO-60 cathode before 1st cycle and after 20th cycle. The sulfur-carbon composite, carbon black and polyvinylidene difluoride (PVDF) are well distributed before 1st cycle in these three cathode. After 20th cycles, the MC/S-MI-60 cathode surface shows a dense solid layer (red circle), but there is no apparent solid layer at the surface of NMC/S-CO-60 cathode. The formation of a dense solid layer on the cathode surface is possibly attributed to gradual aggregation of insulated Li_2_S on the cathode surface, which is one of the main reasons for poor cyclic durability. Due to the excellent confining effect and the fast kinetics of the electrochemical redox reactions, NMC/S-CO-60 cathode can efficient restrain the formation of insulated Li_2_S on the cathode surface.

***Figure S10.*** EIS of NMC/S-CO-60, NMC/S-MI-60 and MC/S-MI-60 cathode before 1st cycle and after 20th cycle. The semi-circle corresponds to the contact and charge transfer resistances, and the interception of the semi-circles and the real axis corresponds to the resistances of the electrolyte. After the extended 20 cycles, (i) the resistance of the electrolyte in MCS-MI-60 cathode is significantly increased possibly due to the dissolved lithium polysulﬁdes increasing the viscosity of the electrolyte and retarding the lithium ion transport, and (ii) the contact and charge transfer resistance of MCS-MI-60 cathode is also changed hugely induced by the formation of an insulating layer of lithium sulﬁde (Li_2_S) as evidenced by figure S9.

***Figure S11.*** EIS profiles of the MCs and NMCs evaluated as electrode materials in the supercapacitors. The NMCs powders were processed into supercapacitor electrodes by mixing them with 5 wt.% polytetra-ﬂuoroethlyene (PTFE), homogenized in a mortar and pestle, then rolled into a thin ﬁlm of uniform thickness, and ﬁnally punched into pellets with a diameter of 13 mm. Electrochemical impedance spectroscopy (EIS) was performed in 3M H_2_SO_4_ using a two-electrode supercapacitor system via an electrochemical working station PCI 4/300 (Gamry Instrument, Warminster, PA, USA). the EIS results of NMCs evaluated as electrode materials in the supercapacitors also suggest the significantly enhanced charge-tranfer kinetics caused by nitrogen doping.


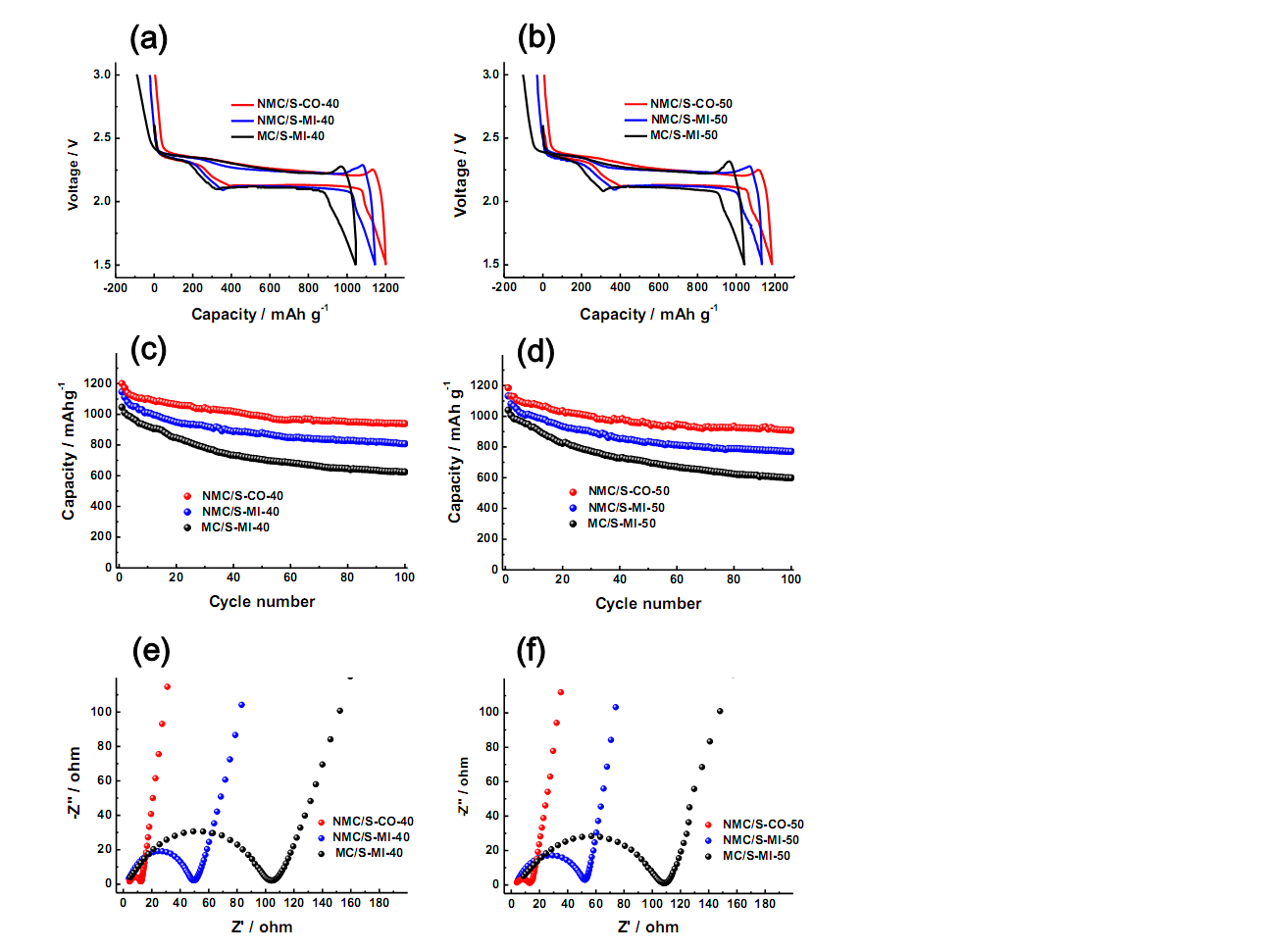


***Figure S12.*** Initial charge-discharge curves of NMC/S series with sulfur loadings of 40 wt.% (a) and 50 wt.% (b); cycle capacity (0.2C) of NMC/S series with sulfur loadings of 40 wt.% (c) and 50 wt.% (d); EIS before 1st cycle of NMC/S series with sulfur loadings of 40 wt.% (e) and 50 wt.% (f).


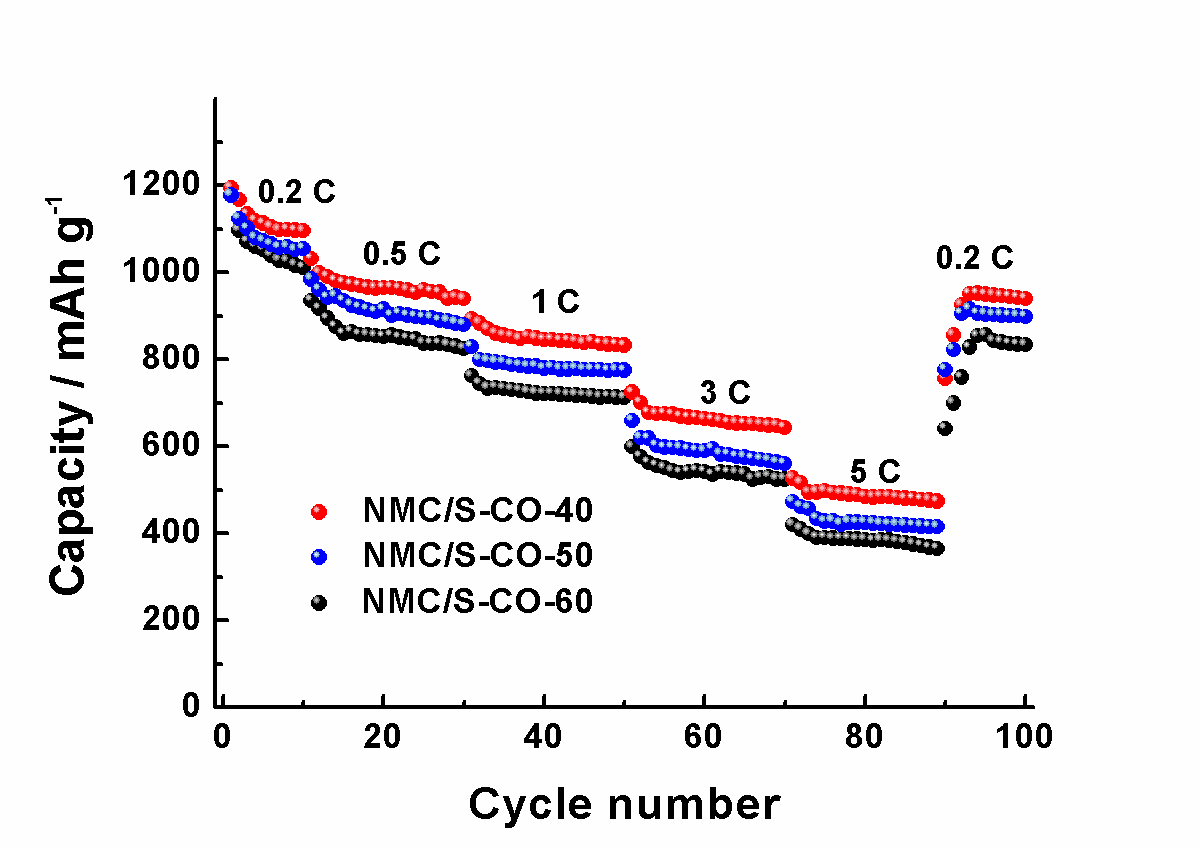


***Figure S13.*** Rate performance of NMC/S-CO series samples. The rate capacity behavior of the NMC/S-CO series composites show that NMC/S-CO-40 is seem to deliver 675 mAh g^-1^ at 3 C (5.1 A g^-1^) and 527 mAh g^-1^ at 5 C (8.5 A g^-1^).
